# Valuation of Go Stimuli or Devaluation of No-Go Stimuli? Evidence of an Increased Preference for Attended Go Stimuli Following a Go/No-Go Task

**DOI:** 10.3389/fpsyg.2017.00474

**Published:** 2017-03-30

**Authors:** Kazuya Inoue, Nobuya Sato

**Affiliations:** ^1^Automotive Human Factors Research Center, Department of Information Technology and Human Factors, National Institute of Advanced Industrial Science and TechnologyTsukuba, Japan; ^2^Department of Psychological Science, Kwansei Gakuin UniversityNishinomiya, Japan

**Keywords:** distractor devaluation, response inhibition, attention, mere exposure effect, preference

## Abstract

Attentional inhibition that occurs during discrimination tasks leads to the negative evaluation of distractor stimuli. This phenomenon, known as the distractor devaluation effect also occurs when go/no-go tasks require response inhibition. However, it remains unclear whether there are interactions between attention and response controls when the distractor devaluation effect occurs. The aims of this study were to investigate whether attention to stimuli in the go/no-go task plays a facilitative role in distractor devaluation through response inhibition, and to clarify whether this effect reflects a decreased preference for no-go stimuli. Participants evaluated the preference for pictures before and after a go/no-go task. In Experiments 1 and 2, they made a go or no-go response depending on the category of pictures displayed (gummy candies or rice crackers), whereas in Experiment 3 they did on the basis digit category, even or odd numbers, superimposed on such pictures. Experiments 1 and 2 demonstrated that the pictures presented as no-go stimuli in the preceding go/no-go task were evaluated as less positive than the pictures presented as go stimuli. This devaluation effect reflected an increased preference for the go stimuli but not a decreased preference for the no-go stimuli. Experiment 3 indicated that response inhibition did not affect the preference for the pictures that had not received attention in a preceding go/no-go task. These results suggest that although attention plays an important role in differential ratings for go and no-go stimuli, such differences, in fact, reflect the valuation of go stimuli.

## Introduction

One’s evaluation of the emotional value of objects appears to be influenced by ways in which an individual interacts with certain objects. For example, people tend to prefer objects that are repeatedly encountered relative to novel (i.e., unexposed) ones. This preferential bias has been labeled as the mere exposure effect (Zajonc, 1968). In addition to this heightening of preference, simply interacting with objects can also lead to a decrease in preference for certain objects (Raymond et al., 2003). In latter study, participants were presented with two different types of Mondrian-like stimuli and they were asked to select one of them as a target based on a specified categorical criterion. In a subsequent rating, participants were required to evaluate the affective valence of these two types of stimuli as well as of novel stimuli. The results showed that the stimuli previously rejected as distractors were subsequently evaluated more negatively than the stimuli selected as target and the novel stimuli. This emotional devaluation of the distractor stimuli, called the distractor devaluation effect (Raymond et al., 2003), is a robust phenomenon that has been observed in various experimental paradigms, such as in a visual search task (Fenske et al., 2004; Raymond et al., 2005), rapid serial visual presentation task (Kihara et al., 2011), and flanker task (Martiny-Huenger et al., 2014). In addition, this effect has been replicated using a wide range of stimuli such as geometric figures (Veling et al., 2008), line drawings (Griffiths and Mitchell, 2008), and human faces (Fenske et al., 2005).

The widely accepted account of the distractor devaluation effect is based on attentional inhibition (Raymond et al., 2003; Fenske and Raymond, 2006; Raymond, 2009). In a typical study of the distractor devaluation effect, participants are asked to ignore distractor stimuli and to select target stimuli. In this situation, it appears that inhibitory processing is involved in the representation of the distractor stimuli, leading to the acquisition of a representational distractor code with an inhibitory status. This inhibitory status is activated and it is ultimately reflected in the subsequent preference rating of a distractor stimulus when it is next encountered. Three findings support this devaluation-by-inhibition account (Raymond, 2009). First, the distractor devaluation effect has been found in experimental paradigms that require participants to inhibit task-unrelated stimuli, such as the visual search (Raymond et al., 2005), negative priming (Goolsby et al., 2009a), and flanker tasks (Martiny-Huenger et al., 2014). Second, the magnitude of the distractor devaluation effect was proportional to the degree of the attentional inhibition, that is, greater attentional inhibition was required, hence more negative emotion was induced to the inhibited stimuli (Raymond et al., 2005). Third, the devaluation-by-inhibition account was also supported by an electrophysiological study (Kiss et al., 2007) as well by the aforementioned behavioral studies. These studies showed that the efficiency of the attentional selection of target stimuli, reflected in the timing of the N2pc component of event-related potentials, was associated with the negative evaluation of distractor stimuli (Kiss et al., 2007).

In addition to the inhibition of attention, response inhibition required in the go/no-go or stop signal tasks also resulted in distractor devaluation (Fenske et al., 2005; Kiss et al., 2008; Veling et al., 2008; Buttaccio and Hahn, 2010; Doallo et al., 2012; Frischen et al., 2012; Wessel et al., 2014). In one of the studies (Kiss et al., 2008), participants were presented with a sequence of stimuli consisting of Asian and Caucasian faces. They were required to respond, by a key press, to the specified category of face (e.g., Asian face; go stimuli), whereas they were asked to refrain from responding to the other category of face (e.g., Caucasian face; no-go stimuli). In a subsequent rating task, participants had to rate the trustworthiness of the faces that had appeared in the preceding go/no-go task. Results indicated that the no-go stimuli were rated less trustworthy than the go-stimuli. The difference was attributed to the decreased trustworthiness for the no-go stimuli but not the increased trustworthiness for the go stimuli because the electrophysiological measure of response inhibition (i.e., nogo-N2) was associated with the negative evaluation of the no-go stimuli. The relationship between emotional devaluation and response inhibition was also confirmed in a neuroimaging study using functional magnetic resonance imaging (Doallo et al., 2012), which demonstrated that the activity in the lateral prefrontal cortex related to response suppression in the go/no-go task was involved in the distractor devaluation effect. However, as these two studies only compared go stimuli with no-go stimuli, they lacked a control condition (e.g., the presentation of unexposed stimuli). Further, several behavioral studies showed that no-go stimuli or stimuli presented with a no-go cue were devaluated when compared with stimuli in the control condition (Fenske et al., 2005; Veling et al., 2008). For example, following the go/no-go task where participants made a go/no-go response depending on the category of a letter superimposed on a picture, they rated the pictures associated with the no-go cue as less attractive than either novel pictures or pictures associated with the go cue (Veling et al., 2008).

Although it has been shown that both attention and response primarily affect the emotional devaluation of stimuli, it remains unclear whether there are interactions between attention and response controls. Specifically, it is unclear whether attention to stimuli has an influence on emotional devaluation based upon (i.e., through) the act of response inhibition. Several studies have demonstrated that stimuli previously presented with no-go cues (such as letter, digit, and colored circle) were devaluated as compared with novel stimuli or stimuli previously presented with go cues (Fenske et al., 2005; Veling et al., 2008; Buttaccio and Hahn, 2010). That is, these studies reported that response inhibition induced the emotional devaluation of unattended and task-irrelevant stimuli. However, to the best of our knowledge, few studies have directly compared the effect of response inhibition on attended (versus unattended) stimuli with respect to devaluations of emotions. In addition, in previous studies the presentation duration of the unattended stimuli was relatively long (about 1000 ms; Fenske et al., 2005; Buttaccio and Hahn, 2010). Therefore, it is possible that a participant’s attention was directed to task-irrelevant stimuli presented concurrently with no-go cues. In summary, it remains unclear whether attention facilitates emotional devaluation through response inhibition.

Previous studies have suggested that attention plays an important role in the modulation of emotional evaluation. For example, the increase in positive affect toward repeatedly presented stimuli, called the mere exposure effect, is more likely to occur for attended stimuli than for unattended stimuli (Yagi et al., 2009; Huang and Hsieh, 2013). Furthermore, attention also plays an important role in evaluative conditioning where neural stimuli (conditioned stimuli), paired with affective stimuli (unconditioned stimuli), acquire the affective valence of the unconditioned stimuli. For example, distracting attention from conditioned and unconditioned stimuli disrupted the formation of conditioned affective valence (Field and Moore, 2012). In addition, distracting attention from the contingency between the conditioned and unconditioned stimuli also disrupted the effect (Kattner, 2012). Given that attention plays an important role in the acquisition of emotional value, emotional devaluation through response inhibition would occur more strongly for attended stimuli than it would for unattended stimuli.

The aim of the present study was to investigate whether attention plays an important role in emotional devaluation through response inhibition. Accordingly, we conducted three experiments to clarify this issue. In Experiments 1 and 2, we examined the effect of response inhibition to attended pictures on their emotional devaluation. As in the previous studies, affective rating was preceded by a go/no-go task (Kiss et al., 2008; Doallo et al., 2012), where participants were asked to make a go or no-go response depending on the category of picture. Following the go/no-go task, participants were asked to report their preference for pictures that had appeared in the go/no-go task. Because the experimental procedure in Experiments 1 and 2 was similar to that of the previous studies (Kiss et al., 2008; Doallo et al., 2012), we predicted that emotional devaluation through response inhibition would occur. That is, the pictures that were previously presented as no-go stimuli would be evaluated less positively than the pictures that were presented as go stimuli. In Experiment 3, we examined the effect of response inhibition on the emotional devaluation of unattended stimuli concurrently presented with no-go cues. In contrast to Experiments 1 and 2, participants were asked to make a go or no-go response depending on the category of a digit target superimposed on the picture stimuli. Following the go/no-go task, participants were asked to rate their preference for the pictures that were (presumably) not attended to in the preceding go/no-go task. If attention plays an important role in the devaluation effect due to response inhibition, emotional devaluation would be attenuated in Experiment 3 compared with that in Experiments 1 and 2. In contrast, if attention is not necessary for emotional devaluation through response inhibition, comparable devaluation effects should be found in all experiments.

An additional important difference between the present and previous studies (Kiss et al., 2008; Doallo et al., 2012) was the inclusion of a control condition. Previous studies only compared the preference for the go stimuli with that for the no-go stimuli. Therefore, it was unclear whether the pictures presented in the go trials acquired positive valence or the pictures presented in the no-go trials acquired negative valence. One possible approach to clarify this is to include novel stimuli that were not presented in the go/no-go task. However, this might be inappropriate given that the distractor devaluation effect can occur in a category-based manner (Goolsby et al., 2009b). That is, it even occurs for novel stimuli belonging to a previously ignored category. Therefore, we did not include novel stimuli for a control condition. Instead, we asked participants to evaluate the preference for the pictures before and after the go/no-go task. This procedure would make it possible to disentangle the effect of response type on emotional devaluation through response inhibition.

## Experiment 1

The aim of Experiment 1 was to investigate the effect of response inhibition to attended pictures on the emotional evaluation of these pictures. To this end, participants were required to complete a go/no-go task, followed by a preference-rating task. In the go/no-go task, participants were asked to press space key when the picture of a specified target category was presented (i.e., a go trial) and to suppress the key response when the picture did not belong to the target category (i.e., a no-go trial). Previous studies have demonstrated that the pictures presented as no-go stimuli were rated less positively in subsequent rating than images presented as go stimuli (Kiss et al., 2008; Doallo et al., 2012). Thus, if attention plays an important role in emotional devaluation through response inhibition, we should obtain the same results as reported in the previous studies (Kiss et al., 2008; Doallo et al., 2012).

### Materials and Methods

#### Ethics Statement

All experiments (Experiments 1, 2, and 3) were approved by the Kwansei Gakuin University Institutional Review Board for Behavioral Research with Human Participants. In the three experiments, written informed consent was obtained from all participants, in accordance with the Declaration of Helsinki.

#### Participants

Thirty adults participated in this experiment (8 males and 22 females; mean age = 19.7 years; *SD* = 1.73). All participants reported normal or corrected-to-normal vision.

#### Stimuli and Apparatus

All stimuli were presented on a 19-inch cathode-ray tube display at a resolution of 1280 × 1024 pixels, at a viewing distance of 52 cm. Stimulus presentation and response collection were controlled by a personal computer running of the Windows XP platform, and using Psychophysics Toolbox extensions (Brainard, 1997; Pelli, 1997) on Matlab (Mathworks Inc.).

**FIGURE 1:**
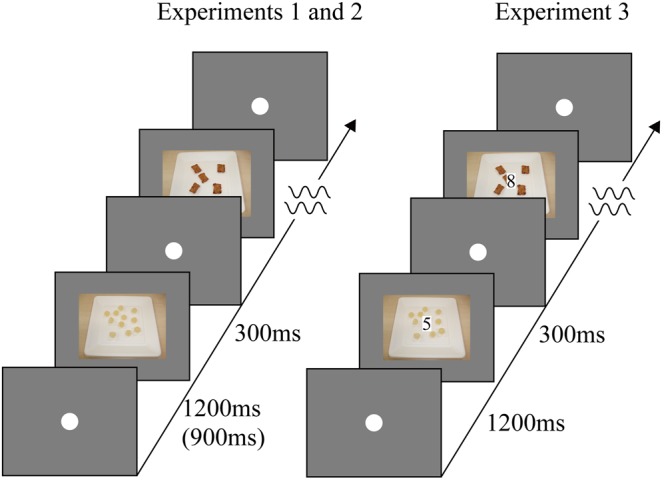
**The stimulus sequence of the go/no-go task in Experiment 1–3.** The digits in parentheses denote the stimulus presentation duration in Experiment 2.

We used pictures of food (gummy candy and rice crackers) to extend the findings of the present study to the control of eating behavior through response inhibition (Houben and Jansen, 2011; Houben et al., 2011, 2012). Similar objects were used because the amount of inhibition necessary to withdraw responses plays an important role in the distractor devaluation effect (Frischen et al., 2012). Forty-five pictures for each category (gummy candy and rice crackers), recorded with a digital camera, were used in Experiment 1 (**Figure 1**). These pictures depicted different types of gummy candy and rice crackers. Five pictures for each category were used in practice trials; the remaining pictures were used in experimental trials. The pictures depicted a handful of gummy candies and rice crackers on a white paper dish. The width and height of each picture were 11.19 and 8.30°, respectively.

#### Procedure

**FIGURE 2:**
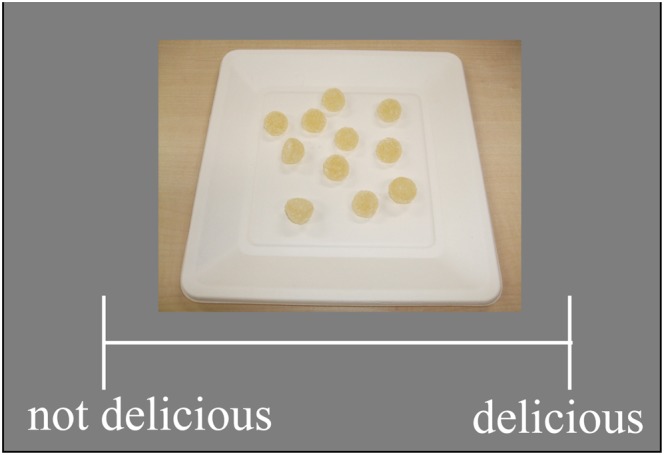
**A sample display of preference ratings.** Participants reported perceived deliciousness by clicking the corresponding position on the visual analog scale.

The experiment began with a preliminary preference-rating task (**Figure 2**). In each trial, a picture of food was presented at the center of the display, along with a visual analog scale (VAS). The height and width of the VAS was 14.91 and 2.06°, respectively. The left end of the VAS indicated that the picture did not look delicious, whereas the right end of the VAS indicated that the picture looked delicious. Participants were asked to report their perceived deliciousness by clicking on a corresponding position on the VAS. The preference-rating task consisted of 10 practice trials (five trials for each food category) followed by 80 experimental trials (40 trials for each category). The presentation order of the pictures was completely randomized.

Following the first preference rating, blocks of go/no-go trials and the second rating trials were administered; the go/no-go and rating blocks were alternately performed, as conducted in the previous studies (Kiss et al., 2008; Doallo et al., 2012). In each trial of the go/no-go task (**Figure 1**), a fixation circle was presented at the center of the display for 1200 ms, followed by the presentation of a picture for 300 ms (i.e., an inter-onset-interval between pictures of 1,500 ms). Participants were asked to press a space key as quickly and accurately as possible when the picture of a target category (gummy candy or rice crackers) was presented and to refrain from a key response when the picture of a non-target category was presented. Although picture duration was 300 ms, participants were allowed to respond during the 1,500 ms preceding the onset of the next picture. The next fixation circle and picture appeared regardless of whether the participant’s response was recorded. Each block of the go/no-go task consisted of 10 trials; 10 pictures, five for each stimulus category, were presented in a random order. The target category was instructed at the start of each block of trials and alternately changed from block to block. That is, in the half of the blocks the pictures of gummy candies were pared with a go response whereas in the remaining blocks the pictures of rice crackers were pared with a go response.

After completion of each go/no-go block of trials, a block of trials requiring preference ratings was presented. Pictures in this second rating task were identical to those in the preceding go/no-go block. The order of the pictures was also the same as that in the go/no-go block. After 10 practice trials for each go/no-go task and rating task, participants performed eight blocks of go/no-go trials and rating trials, respectively. Thus, each block of 10 go/no-go trials was followed by a block of 10 rating trials.

### Results and Discussion

#### Go/No-Go Task

**FIGURE 3:**
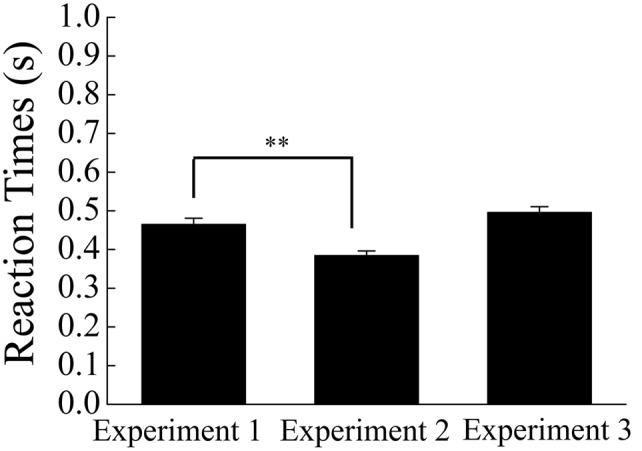
**Mean reaction times for go stimuli as a function of experiments.** Error bars show + 1 SE. Asterisks indicate a significant difference (^∗∗^*p* < 0.01).

**FIGURE 4:**
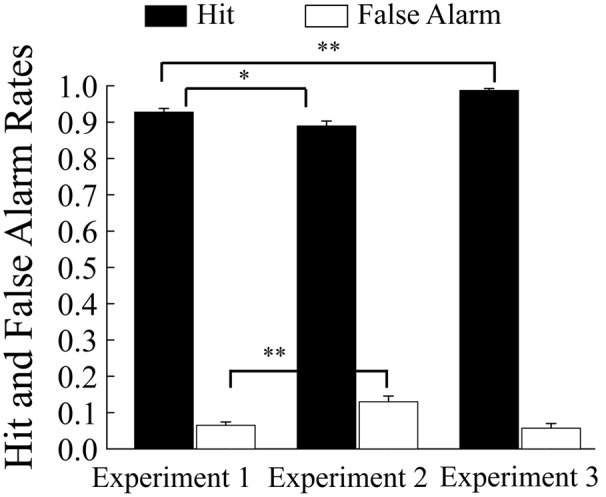
**Mean hit and false alarm rates as a function of experiments.** Error bars show + 1 SE. Asterisks indicate significant differences (^∗^*p* < 0.05, ^∗∗^*p* < 0.01).

Means of reaction time and discrimination performance (hit and false alarm rates) appear in **Figures 3, 4**, respectively.

#### Preference Rating

**FIGURE 5:**
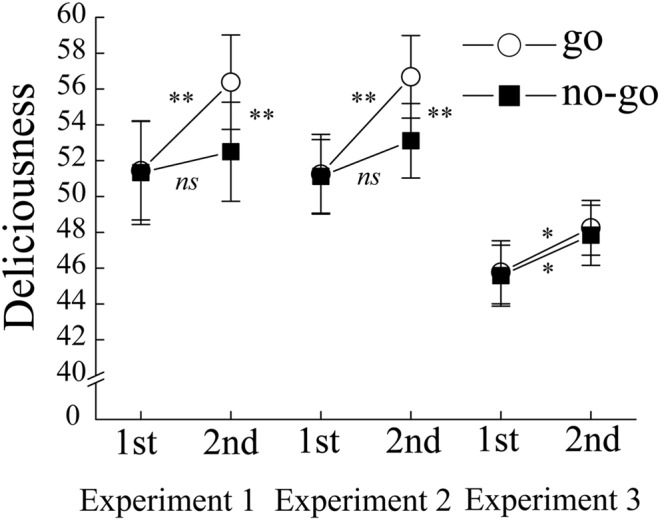
**Mean preference ratings for go and no-go stimuli as a function of rating phase in Experiment 1–3.** White circles indicate the preference for go stimuli whereas black circles indicate the preference for no-go stimuli. Error bars show ± 1 SE. Asterisks indicate significant differences (^∗^*p* < 0.05, ^∗∗^*p* < 0.01).

We excluded the pictures to which the participants made incorrect responses in the go/no-go task from the analysis of preference rating. The mean proportion of excluded pictures was 0.07 (*SE* = 0.01). The responses obtained from the preference task were transformed so that the left end and right ends of the VAS were scored as 0 and 100, respectively. Mean preference rating was calculated in each condition, for each participant (**Figure 5**). An analysis of variance (ANOVA) was carried out on the mean preference ratings with rating phase (first vs. second) and stimulus category (go stimuli vs. no-go stimuli) as within-participant variables. The ANOVA showed significant main effects of rating phase, *F*(1,29) = 10.71, *p* < 0.01, $\eta_{p}^{2}$ = 0.27, and stimulus category, *F*(1,29) = 6.93, *p* < 0.05, $\eta_{p}^{2}$ = 0.19, which was qualified by the interaction between the rating phase and stimulus category, *F*(1,29) = 14.81, *p* < 0.01, $\eta_{p}^{2}$ = 0.34. *Post hoc* simple effects showed that a significant increase in preference for go stimuli was found between the first and second ratings, *F*(1,29) = 16.38, *p* < 0.01, $\eta_{p}^{2}$ = 0.36 whereas there was no significant difference in the preference for no-go stimuli between the first and second ratings, *F*(1,29) = 1.87, *p* = 0.18, $\eta_{p}^{2}$ = 0.06. Importantly, in the second rating the preference for the no-go stimuli was lower than that for the go stimuli, *F*(1,29) = 14.76, *p* < 0.01, $\eta_{p}^{2}$ = 0.34, which is consistent with the results reported in previous studies (Kiss et al., 2008; Doallo et al., 2012). According to the interpretation of these studies, these results suggest that the emotional devaluation of the no-go stimuli occurred through response inhibition (Kiss et al., 2008; Doallo et al., 2012).

Although the response type in the go/no-go task influenced the subsequent preference rating, this effect reflected an increase for the go-stimuli but not a decrease for the no-go stimuli. This outcome appears to be contradictory to the view that emotional evaluation decreases due to response inhibition (Kiss et al., 2008; Doallo et al., 2012). One possible account for a lack of decrease in preference between the first and second ratings is insufficient task difficulty. A previous study found that the magnitude of emotional devaluation is correlated with the amount of inhibition necessary to withdraw responses (Frischen et al., 2012). From this fact, it is possible that emotional devaluation due to response inhibition did not emerge in Experiment 1 because, in this situation, the demand for response inhibition was relatively low because of the insufficient task difficulty. We therefore excluded this possibility in Experiment 2.

## Experiment 2

In Experiment 1, the mean preference rating for the no-go stimuli was comparable in the first and second ratings. One possible cause for the lack of the decrease in preference was that the go/no-go task in Experiment 1 was relatively easy; therefore, it is possible that participants did not need to suppress their response in the go/no-go task. In order to investigate this possibility, the task difficulty in the go/no-go task was increased in Experiment 2 relative to that in Experiment 1. A previous study demonstrated that the shortening of reaction time deadline (i.e., time window to respond) in the go/no-go task increased the task difficulty, which led to the increase in the inhibition necessary to withdraw responses (Benikos et al., 2013). Therefore, in Experiment 2, we used a shorter reaction time deadline to increase the task difficulty in the go/no-go task.

### Methods

#### Participants

Twenty-six adults participated in this experiment (10 males and 16 females; mean age = 19.62 years; *SD* = 1.78). None of them participated in Experiment 1. All participants reported normal or corrected-to-normal vision.

#### Stimuli and Procedure

The stimuli and procedure were identical to those of Experiment 1, except that the duration of the fixation point in the go/no-go task was reduced to 900 ms, to increase the task difficulty. Consequently, the response time deadline was reduced from 1500 ms in Experiment 1 to 1200 ms in Experiment 2.

### Results and Discussion

#### Go/No-Go Task

Mean reaction time has been presented in **Figure 3**. In order to compare the reaction time in Experiment 2 with that in Experiment 1, a one-way ANOVA was conducted with experiments as a between-participant variable. The ANOVA showed that the mean reaction time was shorter in Experiment 2 than in Experiment 1, *F*(1,54) = 16.47, *p* < 0.01, $\eta_{p}^{2}$ = 0.23, indicating that participants were urged to respond quickly by the reduction of the response time deadline. Mean hit and false alarm rates have been shown in **Figure 4**. In order to compare the task difficulty in Experiment 2 with that in Experiment 1, a one-way ANOVA was conducted on the hit rates with experiments as a between-participant variable. The ANOVA showed that the mean hit rate was lower in Experiment 2 than in Experiment 1, *F*(1,54) = 5.12, *p* = 0.03, $\eta_{p}^{2}$ = 0.09. In contrast, the ANOVA conducted on the false alarm rates showed that the mean false alarm rate was higher in Experiment 2 than in Experiment 1, *F*(1,54) = 13.26, *p* < 0.01, $\eta_{p}^{2}$ = 0.20. These results suggest that the reduction of the response time deadline in the go/no-go task increased the task difficulty in Experiment 2 compared with that in Experiment 1.

#### Preference Rating

As in Experiment 1, we excluded the pictures presented in the error trials from the following analysis. The mean proportion of excluded pictures was 0.12 (*SE* = 0.01). The same ANOVA used in Experiment 1 was used to analyze data of Experiment 2. The resulting analysis revealed a significant main effect of rating phase, *F*(1,25) = 8.85, *p* < 0.01, $\eta_{p}^{2}$ = 0.26, but a non-significant effect of stimulus category, *F*(1,25) = 2.43, *p* = 0.13, $\eta_{p}^{2}$ = 0.09. As in Experiment 1, the interaction between rating phase and stimulus category was significant in the present experiment, *F*(1,25) = 13.38, *p* < 0.01, $\eta_{p}^{2}$ = 0.35. *Post hoc* simple effects showed that the go stimuli were rated more positively in the second rating than in the first rating, *F*(1,25) = 16.02, *p* < 0.01, $\eta_{p}^{2}$ = 0.39, whereas the preference for the no-go stimuli showed no differences between the first and second rating phases, *F*(1,25) = 2.29, *p* = 0.14, $\eta_{p}^{2}$ = 0.08. Furthermore, in the second rating task, the no-go stimuli were rated more negatively than the go stimuli, *F*(1,25) = 7.86, *p* < 0.01, $\eta_{p}^{2}$ = 0.21. These results clearly replicate the results of Experiment 1, suggesting that the response type in the go/no go task influenced the preference rating in the second rating phase. As in Experiment 1, mean preference ratings for the no-go stimuli did not differ between the no-go first and second rating phases. Thus, the lack of decrease in the preference for the no-go stimuli could not be attributed to the insufficient task difficulty in the go/no-go task because task difficulty in Experiment 2 was greater than that in Experiment 1.

To compare devaluation effects between Experiments 1 and 2, we calculated devaluation scores by subtracting the mean preference score for no-go stimuli in the second rating from that for go stimuli in the second rating for each participant. The means of the devaluation scores were 3.88 (*SE* = 1.01) in Experiment 1 and 3.57 (*SE* = 1.39) in Experiment 2. Then, these mean values were compared using a one way ANOVA, showing non-significant effect of experiments, *F*(1,54) = 0.03, *p* = 0.86, $\eta_{p}^{2}$ = 0.00. That is, increasing the task difficulty did not affect the devaluation effect through response inhibition, which appears to be contradictory to the inhibitory account (Raymond et al., 2003; Fenske and Raymond, 2006; Raymond, 2009).

## Experiment 3

The results of Experiments 1 and 2 showed that the type of response influenced the preference for the pictures to which attention was directed in the preceding go/no-go task. Experiment 3 was designed to investigate whether the type of response also modulates the preference for unattended pictures in the preceding go/no-go task. In contrast to Experiments 1 and 2, in Experiment 3, participants were required to discriminate whether a digit superimposed on a picture was either odd or even. Because participant’s attention was focused on the target digit, it is plausible to consider that their attention is less directed to the pictures of gummy candies and rice crackers in the go/no-go task compared with that in Experiments 1 and 2. Thus, if attention plays an important role in emotional devaluation through response inhibition, the devaluation effect should be attenuated in Experiment 3 relative to that in Experiments 1 and 2.

### Methods

#### Participants

Twenty-eight adults participated in this experiment (6 males and 22 females; mean age = 20.36 years; *SD* = 3.88). None of them participated in Experiments 1 or 2. All participants reported normal or corrected-to-normal vision.

#### Stimuli and Procedure

The stimuli and the procedure were identical to those of Experiment 1, with the following exceptions. In the go/no-go task, the pictures of gummy candies and rice crackers were sequentially presented as in Experiment 1. However, a digit depicted on a white placeholder (0.39 and 0.77° in width and height, respectively) was superimposed on each picture (**Figure 1**). Odd numbers (1, 3, 5, 7, or 9) were presented in half of the trials in a block, whereas even numbers (0, 2, 4, 6, or 8) were presented in the other half of the trials in the block. In a half of the blocks, participants were required to press a space key when an odd number was presented, whereas they were required to refrain from the response when an even number was presented. In the other half of blocks, the assignment of the target category to the type of response was reversed. The target category was alternately changed from block to block and the order was counterbalanced across participants. The combination of the category of digits and the category of pictures was fixed for a participant. For example, one participant, only odd numbers (or only even numbers) were presented on the pictures of gummy candies (or only pictures of rice crackers). This combination was counterbalanced across participants.

### Results and Discussion

#### Go/No-Go Task

Mean reaction time has been shown in **Figure 3**. In order to compare the reaction time in Experiment 3 with that in Experiment 1, a one-way ANOVA was conducted with experiments as a between-participant variable. However, the ANOVA did not reach significance, *F*(1,56) = 2.10, *p* = 0.15, $\eta_{p}^{2}$ = 0.04. Mean hit and false alarm rates have been shown in **Figure 4**. In order to compare the task difficulty in Experiment 3 with that in Experiment 1, an ANOVA was conducted on the hit and false alarm rates, with experiments (Experiment 1 vs. Experiment 3) as a between-participant variable. The analysis revealed that the mean hit rate was higher in Experiment 3 than in Experiment 1, *F*(1,56) = 26.00, *p* < 0.01, $\eta_{p}^{2}$ = 0.32, whereas the mean false alarm rate did not differ between experiments, *F*(1,56) = 0.25, *p* = 0.62, $\eta_{p}^{2}$ = 0.004.

The difference observed between the experiments cannot be attributed to a difference in difficulty due to the timing of stimuli associated with reduced stimulus durations, because both stimulus durations and response time deadlines were identical in Experiments 1 and 3. Instead, this outcome is likely to reflect the difference in the discriminability of the target and distractor stimuli in these two experiments. In Experiment 1, several pictures of gummy candies (rice crackers) were considerably similar to the pictures in the other category; therefore, participants misidentified the pictures of one category as those of the other category. In contrast, the discrimination between even and odd numbers was relatively easy, as indicated by the higher hit rate in Experiment 3 than that in Experiment 2. From this, and from the absence of differences in the false alarm rates and the mean reaction times, it is plausible to consider that the demand for response inhibition in Experiment 3 was not much different from that in Experiment 1.

#### Preference Rating

As in Experiment 1, we excluded the pictures presented in the error trials from the following analysis. The mean proportion of the excluded pictures was 0.03 (*SE* = 0.01). Mean preference ratings have been shown in **Figure 5**. The same ANOVA as in Experiment 1 was conducted on the preference ratings of Experiment 3. The ANOVA showed a significant main effect of rating phase (first vs. second), *F*(1,27) = 8.16, *p* < 0.001, $\eta_{p}^{2}$ = 0.23, indicating that the mean preference score was higher in the second rating phase than that in the first rating phase. This effect probably reflects the mere exposure effect (Zajonc, 1968). In contrast to Experiment 1, there was neither main effect of stimulus category, *F*(1,27) = 0.21, *p* = 0.65, $\eta_{p}^{2}$ = 0.008, nor an interaction between rating phase and stimulus category, *F*(1,27) = 0.06, *p* = 0.81, $\eta_{p}^{2}$ = 0.002, indicating that emotional devaluation through response inhibition was not evident in Experiment 3. These results suggest that, in Experiment 3, the type of response in the go/no-go task did not affect the preference rating for the picture that had not been attended in the go/no-go task.

To ascertain the importance of attention in the devaluation effect through response inhibition, we compared the mean devaluation scores between Experiments 1 and 3 using a one-way ANOVA with experiments as a between-participants variable, which indicated that the mean devaluation score was greater in Experiment 1 (*M* = 3.88, *SE* = 1.01) than that in Experiment 3 (*M* = 0.41, *SE* = 0.84), *F*(1,56) = 6.86, *p* = 0.01, $\eta_{p}^{2}$ = 0.11. This result suggests that attention to the stimuli in the go/no-go task facilitate emotional devaluation through response inhibition.

## General Discussion

The aim of the present study was to investigate whether attention plays an important role in emotional devaluation through response inhibition. In addition, another aim was to clarify whether the devaluation through response inhibition reflected a decreased preference for no-go stimuli or an increased preference for go stimuli. To this end, we assessed the effect of response inhibition in a go/no-go task on subsequent rating of preference for the pictures presented. In Experiment 1, participants made a go or no-go response depending on the category of picture stimuli, which was preceded and followed by a preference rating task for the picture presented in the go/no-go task. The results showed that, in the second rating, no-go stimuli (i.e., previously rejected stimuli) were rated less positively than go-stimuli (i.e., previously accepted stimuli). This finding is consistent with those of previous studies that demonstrated that response inhibition results in the emotional devaluation of the no-go stimuli (Kiss et al., 2008; Doallo et al., 2012). The results of Experiment 1 were replicated in Experiment 2, where the task difficulty of the go/no-go task was increased by reducing the reaction time deadline. In contrast to Experiments 1 and 2, Experiment 3 revealed that such a decrease from response inhibition was not obtained when the participants made a go/no-go response depending on the category of a digit superimposed on a depiction of the stimulus. In summary, we found that different ratings between the go and no-go stimuli occurred only when the participants attended to the picture stimuli in the go/no-go task.

Although an apparent devaluation effect occurred when participant’s attention was directed to the picture stimuli, this devaluation effect really reflected an increased preference for the go stimuli and not a decreased preference for the no-go stimuli. That is, we found no real devaluation effect through response inhibition, which is inconsistent with previous studies suggesting that response inhibition results in the devaluation of no-go stimuli (Kiss et al., 2008; Doallo et al., 2012). Note that these studies used a similar procedure as that in the present study but they lacked a control condition. They only compared the go stimuli with the no-go stimuli following a go/no-go task. Therefore, it is plausible that the valuation of the go stimuli but not the devaluation of the no-go stimuli occurs when the blocks of go/no-go trials and rating trials are conducted alternately, as in this and previous studies (Kiss et al., 2008; Doallo et al., 2012). We do not suggest that devaluation through response inhibition does not occur in any situation (Veling et al., 2008). However, it is suggested that in certain situations different ratings between go and no-go stimuli might be attributed to the increased preference for attended go stimuli.

One may think that insufficient task difficulty in the go/no-go task explains the lack of the difference in preference for the no-go stimuli between the first and second ratings. More specifically, the demand for inhibitory control might not be sufficient if the go/no-go task is relatively easy for participants. However, in Experiment 2, participants’ preference for the no-go stimuli did not differ between the first and second ratings, despite the fact that task difficulty in the go/no-go task was greater than that in Experiment 1. In addition, false alarm rate, which is one of the indexes of task difficulty, was equivalent or higher in the present study than were those reported in previous studies that have demonstrated emotional devaluation due to response inhibition (Kiss et al., 2008; Doallo et al., 2012). Therefore, the results of Experiments 1 and 2 suggest that the lack of the difference in preference for the no-go stimuli between the first and second rating could not be attributed to the insufficient task difficulty in the go/no-go task.

Why did the preference for go stimuli increase from the first to second ratings when the participants attended to the picture stimuli in the go/no-go task? One possible explanation, which is contrary to the inhibitory account (Kiss et al., 2008; Doallo et al., 2012) is that preference ratings are affected by the execution, but not by the inhibition of a response. In fact, a recent study reported that items previously presented with a tone were more often selected as more preferable than items presented without the tone when participants were required to respond to the tone. However, this selection bias was not observed when participants were required to inhibit responses on hearing the tone (Schonberg et al., 2014). Assuming that go responses are viewed as approaching responses to go stimuli, it is possible that the stimuli paired with the go response acquired positive valence, as reported in a previous study indicating that stimuli paired with an approaching response acquired positive valence (Cacioppo et al., 1993). Consistent with this account, the preference for the go stimuli increased from the first to second ratings. According to this explanation, the lack of differences in the second preference rating (Experiment 3 in the present study) might indicate the importance of attention in associating the execution of the response with the stimuli presented at the response, which is similar to the importance of attention in the association between multiple stimuli (Field and Moore, 2012; Kattner, 2012). In addition, this account might be consistent with the result that increased effort to inhibit responses did not affect preference ratings (Experiment 2 in the present study).

Another possibility is that increased preference for go stimuli could be attributed to the attentional modulation of the mere exposure effect. Previous studies have shown that attention to stimuli facilitate the mere exposure effect to those stimuli (Yagi et al., 2009; Huang and Hsieh, 2013). Given this finding and the increased preference for go stimuli, it could be inferred that participant’s attention was directed toward the go stimuli for a longer duration than to the no-go stimuli because of the additional time required to respond to the go stimuli, which might have resulted in the increased preference for the go stimuli. Consistent with this possibility, the increased preference from the first to second ratings was comparable between the go and no-go stimuli when participant’s attention was distracted from the pictures themselves (Experiment 3). The present study was not designed to determine if attention, or response execution played a critical role in the increased preference for the go stimuli. Nevertheless, an important implication of the present study is that devaluation through response inhibition does not occur under certain situations.

Finally, the results of the present study cautions us regarding the concept of devaluation due to response inhibition and its application to problems such as eating disorders. For example, recent studies have shown that response inhibition results in decreased food consumption (Houben and Jansen, 2011; Houben et al., 2011, 2012). For example, the consumption of chocolates was reduced after participants suppressed a key response when a picture of chocolates was presented under the no-go stimulus (Houben and Jansen, 2011). In addition, response inhibition also reduced the consumption of beer (Houben et al., 2011, 2012). Contrary to the explanation of these results presented in previous studies, the present study demonstrated that response execution and not response inhibition increased the preference for a stimulus. Therefore, it is possible that under certain situations, response execution increases the consumption of suitable foods. Further studies are needed to clarify the exact conditions under which response inhibition results in the emotional devaluation of no-go stimuli.

## Author Contributions

KI and NS designed this study. KI performed the experiments and analyzed the data. KI and NS wrote the manuscript.

## Conflict of Interest Statement

The authors declare that the research was conducted in the absence of any commercial or financial relationships that could be construed as a potential conflict of interest. The reviewer EDR and handling Editor declared their shared affiliation, and the handling Editor states that the process nevertheless met the standards of a fair and objective review.

## References

[B1] Benikos N. Johnstone S. J. Roodenrys S. J. (2013). Varying task difficulty in the Go/No-go task: the effects of inhibitory control, arousal, and perceived effort on ERP components. *Int. J. Psychophysiol.* 87 262–272. 10.1016/j.ijpsycho.2012.08.005 22902315

[B2] Brainard D. H. (1997). The psychophysics toolbox. *Spat. Vis.* 10 433–436. 10.1163/156856897X00357 9176952

[B3] Buttaccio D. R. Hahn S. (2010). The effect of behavioral response on affective evaluation. *Acta Psychol.* 135 343–348. 10.1016/j.actpsy.2010.09.004 20937503

[B4] Cacioppo J. T. Priester J. R. Bernston G. G. (1993). Rudimentary determinants of attitudes. II: arm flexion and extension have differential effects on attitudes. *J. Pers. Soc. Psychol.* 65 5–17. 10.1037/0022-3514.65.1.5 8355142

[B5] Doallo S. Raymond J. E. Shapiro K. L. Kiss M. Eimer M. Nobre A. C. (2012). Response inhibition results in the emotional devaluation of faces: neural correlates as revealed by fMRI. *Soc. Cogn. Affect. Neurosci.* 7 649–659. 10.1093/scan/nsr031 21642353 PMC3427860

[B6] Fenske M. J. Raymond J. E. (2006). Affective Influences of selective attention. *Curr. Dir. Psychol. Sci.* 15 312–316. 10.1111/j.1467-8721.2006.00459.x

[B7] Fenske M. J. Raymond J. E. Kessler K. Westoby N. Tipper S. P. (2005). Attentional inhibition has social-emotional consequences for unfamiliar faces. *Psychol. Sci.* 16 753–758. 10.1111/j.1467-9280.2005.01609.x 16181435

[B8] Fenske M. J. Raymond J. E. Kunar M. A. (2004). The affective consequences of visual attention in preview search. *Psychon. Bull. Rev.* 11 1055–1061. 10.3758/BF03196736 15875975

[B9] Field A. P. Moore A. C. (2012). Dissociating the effects of attention and contingency awareness on evaluative conditioning effects in the visual paradigm. *Cogn. Emot.* 19 217–243. 10.1080/02699930441000292 22686602

[B10] Frischen A. Ferrey A. E. Burt D. H. R. Pistchik M. Fenske M. J. (2012). The affective consequences of cognitive inhibition: devaluation or neutralization? *J. Exp. Psychol. Hum. Percept. Perform.* 38 169–179. 10.1037/a0025981 22022896

[B11] Goolsby B. A. Shapiro K. L. Raymond J. E. (2009a). Distractor devaluation requires visual working memory. *Psychon. Bull. Rev.* 16 133–138. 10.3758/PBR.16.1.133 19145023

[B12] Goolsby B. A. Shapiro K. L. Silvert L. Kiss M. Fragopanagos N. Taylor J. G. (2009b). Feature-based inhibition underlies the affective consequences of attention. *Vis. Cogn.* 17 500–530. 10.1080/13506280801904095

[B13] Griffiths O. Mitchell C. J. (2008). Negative priming reduces affective ratings. *Cogn. Emot.* 22 1119–1129. 10.1080/02699930701664930

[B14] Houben K. Havermans R. C. Nederkoorn C. Jansen A. (2012). Beer à no-go: learning to stop responding to alcohol cues reduces alcohol intake via reduced affective associations rather than increased response inhibition. *Addiction* 107 1280–1287. 10.1111/j.1360-0443.2012.03827.x 22296168

[B15] Houben K. Jansen A. (2011). Training inhibitory control. A recipe for resisting sweet temptations. *Appetite* 56 345–349. 10.1016/j.appet.2010.12.017 21185896

[B16] Houben K. Nederkoorn C. Wiers R. W. Jansen A. (2011). Resisting temptation: decreasing alcohol-related affect and drinking behavior by training response inhibition. *Drug Alcohol Depend.* 116 132–136. 10.1016/j.drugalcdep.2010.12.011 21288663

[B17] Huang Y.-F. Hsieh P.-J. (2013). The mere exposure effect is modulated by selective attention but not visual awareness. *Vision Res.* 91 56–61. 10.1016/j.visres.2013.07.017 23933239

[B18] Kattner F. (2012). Revisiting the relation between contingency awareness and attention: evaluative conditioning relies on a contingency focus. *Cogn. Emot.* 26 166–175. 10.1080/02699931.2011.565036 21557120

[B19] Kihara K. Yagi Y. Takeda Y. Kawahara J. I. (2011). Distractor devaluation effect in the attentional blink: direct evidence for distractor inhibition. *J. Exp. Psychol. Hum. Percept. Perform.* 37 168–179. 10.1037/a0019948 20731524

[B20] Kiss M. Goolsby B. A. Raymond J. E. Shapiro K. L. Silvert L. Nobre A. C. (2007). Efficient attentional selection predicts distractor devaluation: event-related potential evidence for a direct link between attention and emotion. *J. Cogn. Neurosci.* 19 1316–1322. 10.1162/jocn.2007.19.8.1316 17651005 PMC2397542

[B21] Kiss M. Raymond J. E. Westoby N. Nobre A. C. Eimer M. (2008). Response inhibition is linked to emotional devaluation: behavioural and electrophysiological evidence. *Front. Hum. Neurosci.* 2:13 10.3389/neuro.09.013.2008 PMC2572209 18958213

[B22] Martiny-Huenger T. Gollwitzer P. M. Oettingen G. (2014). Distractor devaluation in a flanker task: object-specific effects without distractor recognition memory. *J. Exp. Psychol. Hum. Percept. Perform.* 40 613–625. 10.1037/a0034130 24016067

[B23] Pelli D. G. (1997). The Videotoolbox software for visual psychophysics: transforming numbers into movies. *Spat. Vis.* 10 437–442. 10.1163/156856897X00366 9176953

[B24] Raymond J. (2009). Interactions of attention, emotion and motivation. *Prog. Brain Res.* 176 293–308. 10.1016/S0079-6123(09)17617-3 19733764

[B25] Raymond J. E. Fenske M. J. Tavassoli N. T. (2003). Selective attention determines emotional responses to novel visual stimuli. *Psychol. Sci.* 14 537–542. 10.1046/j.0956-7976.2003.psci_1462.x 14629683

[B26] Raymond J. E. Fenske M. J. Westoby N. (2005). Emotional devaluation of distracting patterns and faces: a consequence of attentional inhibition during visual search? *J. Exp. Psychol. Hum. Percept. Perform.* 31 1404–1415. 10.1037/0096-1523.31.6.1404 16366798

[B27] Schonberg T. Bakkour A. Hover A. M. Mumford J. A. Nagar L. Perez J. (2014). Changing value through cued approach: an automatic mechanism of behavior change. *Nat. Neurosci.* 17 625–630. 10.1038/nn.3673 24609465 PMC4041518

[B28] Veling H. Holland R. W. van Knippenberg A. (2008). When approach motivation and behavioral inhibition collide: behavior regulation through stimulus devaluation. *J. Exp. Soc. Psychol.* 44 1013–1019. 10.1016/j.jesp.2008.03.004

[B29] Wessel J. R. O’Doherty J. P. Berkebile M. M. Linderman D. Aron A. R. (2014). Stimulus devaluation induced by stopping action. *J. Exp. Psychol. Gen.* 143 2316–2329. 10.1037/xge0000022 25313953 PMC4244281

[B30] Yagi Y. Ikoma S. Kikuchi T. (2009). Attentional modulation of the mere exposure effect. *J. Exp. Psychol. Learn. Mem. Cogn.* 35 1403–1410. 10.1037/a0017396 19857012

[B31] Zajonc R. B. (1968). Attitudinal effects of mere exposure. *J. Pers. Soc. Psychol.* 9 1–27. 10.1037/h00258485667435

